# Data-Driven Suboptimal Scheduling of Switched Systems

**DOI:** 10.3390/s20051287

**Published:** 2020-02-27

**Authors:** Chi Zhang, Minggang Gan, Jingang Zhao, Chenchen Xue

**Affiliations:** State Key Laboratory of Intelligent Control and Decision of Complex Systems, School of Automation, Beijing Institute of Technology, Beijing 100081, China; zhangchibit@foxmail.com (C.Z.); zhaojingang521@126.com (J.Z.); xuechchcalt@163.com (C.X.)

**Keywords:** optimal switching, data-driven control, switched systems, policy iteration, continuous time

## Abstract

In this paper, a data-driven optimal scheduling approach is investigated for continuous-time switched systems with unknown subsystems and infinite-horizon cost functions. Firstly, a policy iteration (PI) based algorithm is proposed to approximate the optimal switching policy online quickly for known switched systems. Secondly, a data-driven PI-based algorithm is proposed online solely from the system state data for switched systems with unknown subsystems. Approximation functions are brought in and their weight vectors can be achieved step by step through different data in the algorithm. Then the weight vectors are employed to approximate the switching policy and the cost function. The convergence and the performance are analyzed. Finally, the simulation results of two examples validate the effectiveness of the proposed approaches.

## 1. Introduction

Switched systems consisting of several subsystems and a switching policy ruling the switching among them [1,2] arise in certain application situations [3,4] such as a system which has to collect data sequentially from a number of sensory sources and switches its attention among the data sources [5,6]. The switching among subsystems complicates the control problems and many of the problems remain to be open such as the optimal control problems. Optimal control [7,8] problems of switched systems have attracted considerable attention over the past few years. Thereinto, the optimal scheduling problem of switched systems is investigated in this paper.

Multiple approaches have been introduced to solve the optimal control problems for switched systems. Gradient-based approaches are investigated to solve the optimal switching time problems [9] and optimal scheduling problems [10,11] directly, usually in a finite time horizon. By utilizing control inputs to represent the switching policy, embedding approaches transform the optimal control problems of switched systems to traditional optimal control problems to address [12,13,14]. Adaptive dynamic programming (ADP) [15] approaches are introduced to solve the optimal control problems for switched systems with different initial conditions directly [16,17,18,19,20,21,22]. For optimal scheduling of switched systems with infinite-horizon cost functions, ADP approaches perform well to provide approximate global optimal solutions directly.

Approximate global optimal solutions are derived in feedback forms through ADP for optimal scheduling of switched systems with discrete-time dynamics and finite-horizon cost functions [17] or infinite-horizon cost functions [18]. Then further research is conducted for problems with switching cost [19] or state jumps [20]. For optimal scheduling of switched systems with continuous-time dynamics, an approximate feedback solution is proposed based on policy iteration (PI) algorithm with its offline, online, and concurrent implementation [21]. Then, a PI algorithm with recursive least squares is proposed and modified into a single loop PI algorithm to reduce the computational burden [22].

The aforementioned optimal control approaches are deduced based on the a priori knowledge of system models. However, not all system models can be completed acquired so that approaches independent of system models [23,24,25] require investigated. For this purpose, some model-free optimal control approaches have been studied for switched systems. Adaptive dynamic programming approaches are presented respectively for a continuous-time switched system with an infinite-horizon cost function [26] and a discrete-time switched system with a finite-horizon cost function [27] under the assumption that dynamic equations can be evaluated at some sets. Gradient-decent approaches only employing state data are proposed to solve optimal switching problems for continuous-time switched systems with finite-horizon cost functions [28,29]. Data-driven research utilizes real-life data measured by sensory sources to achieve the intrinsic information of systems [30] and can solve the problems of switched systems with unknown subsystems, such as the data-driven framework for discovering cyber-physical systems directly from the data [31]. Thereinto, data-driven ADP approaches provide possible solutions for optimal control problems of switched systems [32,33,34] and based on that, the following approach is designed to adapt to the complexity of switched systems which is brought by switching. Then in this paper, the data-driven optimal scheduling approach is investigated for continuous-time switched systems with unknown subsystems. At first, an online PI-based algorithm inspired by the off-policy learning method [35,36] is proposed to approximate the optimal solution quickly for optimal scheduling problems with known system models first and based on that, a data-driven PI-based algorithm is formulated for optimal scheduling of continuous-time switched systems with infinite-horizon cost functions, which don’t require that dynamic equations can be evaluated or known at some sets. Moreover, common online algorithms usually keep collecting data with the updating switching policies applied to the system at each iteration while the online algorithms in this paper take advantages of the data produced only by the initial switching policy.

The contribution of this paper is stated as follows: (1) with the data produced by the initial switching policy, an online PI-based algorithm is proposed to approximate the optimal solution quickly for optimal scheduling of known switched systems. (2) A data-driven PI-based algorithm is designed to solve optimal scheduling problems for switched systems with infinite-horizon cost functions and unknown subsystems, solely from the data produced by the initial switching policy, which has not been achieved well in existing literature as far as we know. (3) The convergence is proved and the optimality is analyzed.

The remainder of the paper is organized as follows. In Section 2, the problem is stated and the classic PI approach is introduced for the optimal scheduling problem. In Section 3, online PI-based algorithms are proposed for switched systems with known subsystems and unknown subsystems. In Section 4, simulation results are shown to indicate the effectiveness of the algorithms. Finally, the conclusion is drawn in Section 5.

## 2. Preliminaries

Consider the switched system as follows:

(1) $$\dot{x}\left(t\right)=f_{v}\left(x\left(t\right)\right),x\left(0\right)=x_{0},$$

where $x\left(t\right)\in R^{n}$ is the system state, v∈V represents the index of the active subsystem, V={1,2,…,N} is the index set of all subsystems, *N* is the number of subsystems, and $f_{v}:R^{n}\to R^{n}$ denotes the unknown dynamics of subsystem *v*. $f_{v}\left(v\in V\right)$ is Lipschitz continuous in Ω where $\Omega \subset R^{n}$ including the origin is the region of interest and there exists v∈V such that $f_{v}\left(0\right)=0$.

In this paper, the problem to be addressed consists in seeking out the optimal switching policy $v^{*}$ to minimize the following cost function:

(2) $$J=\int_{0}^{\infty }Q(x(\tau ))d\tau ,$$

where $Q:R^{n}\to R$ is a positive definite function. During the time interval, the cost-to-go from the time *t* to infinity with the state x(t) at time *t* can be described as [37]:

(3) $$V\left(x\left(t\right)\right)=\int_{t}^{\infty }Q(x(\tau ))d\tau$$

and then

(4) $$V\left(x\left(t\right)\right)=\int_{t}^{t+\delta t}Q(x(\tau ))d\tau +V\left(x\left(t+\delta t\right)\right).$$

For the optimal problem, the admissible switching policy should be introduced and the relevant assumption is made as [22].

**Definition** **1.** *For system (1), a switching policy is called admissible with respect to the cost (2), if it stabilizes the system in *Ω* and for all $x_{0}\in \Omega$, the cost $V(x_{0})$ is finite.*

**Assumption** **1.** *There exists at least one admissible policy for the system.*

According to the Bellman principle of optimality, the optimal cost-to-go can be represented by

(5) $$V^{*}\left(x\left(t\right)\right)=\min_{v}\left(\int_{t}^{t+\delta t}Q(x(\tau ))d\tau +V^{*}\left(x\left(t+\delta t\right)\right)\right),$$

and when δt→0, the corresponding optimal switching policy $v^{*}$ can be represented in a state-feedback form:

(6) $$v^{*}\left(x\right)=\underset{v}{arg\min}\left(\int_{t}^{t+\delta t}Q(x(\tau ))d\tau +V^{*}\left(x\left(t+\delta t\right)\right)\right).$$

When δt→0, with first-order Taylor expansion of $V^{*}\left(x\left(t+\delta t\right)\right)$ applied in (5) and (6), the HJB equation can be given as [21]:

$$\min_{v(x)}\left(Q\left(x\right)+{\left(\frac{\partial V^{*}\left(x\right)}{\partial x}\right)}^{T}f_{v(x)}\left(x\right)\right)=Q\left(x\right)+{\left(\frac{\partial V^{*}\left(x\right)}{\partial x}\right)}^{T}f_{v^{*}\left(x\right)}\left(x\right)=0,$$

where x=x(t), $\frac{\partial (\cdot )}{\partial x}={\left[\frac{\partial (\cdot )}{\partial x_{1}},\frac{\partial (\cdot )}{\partial x_{2}},\ldots ,\frac{\partial (\cdot )}{\partial x_{n}}\right]}^{T}$ when (·) is a scalar and the corresponding optimal switching policy $v^{*}$ is given as [21]:

$$v^{*}\left(x\right)=\underset{i\in V}{arg\min}\left({\left(\frac{\partial V(x)}{\partial x}\right)}^{T}f_{i}\left(x\right)\right).$$

Then, a PI approach can be applied to solve the optimal scheduling problem. Given an admissible switching policy $v^{0}$, the PI approach [21,22] is stated as follows:

(7) $$Q\left(x\right)+{\left(\frac{\partial V^{k}\left(x\right)}{\partial x}\right)}^{T}f_{v^{k}\left(x\right)}\left(x\right)=0$$

(8) $$v^{k+1}\left(x\right)=\underset{i\in V}{arg\min}\left({\left(\frac{\partial V^{k}\left(x\right)}{\partial x}\right)}^{T}f_{i}\left(x\right)\right),$$

where *k* is the iteration number. The cost-to-go is solved in policy evaluation (7) and the switching policy is updated in policy improvement (8) iteratively. The stability and convergence are stated in the following theorem which has been proved in [21,38].

**Theorem** **1.** *For the system (1) with the cost (2), if the value function sequence ${\left\{V^{k}\right\}}_{k=0}^{\infty }$ and the switching policy sequence ${\left\{v^{k}\right\}}_{k=0}^{\infty }$ are generated through (7) and (8) initiating from a stabilizing initial policy $v^{0}$, the value functions ${\left\{V^{k}\right\}}_{k=0}^{\infty }$ converge to the optimal value function $V^{*}$, and the switching policies ${\left\{v^{k}\right\}}_{k=0}^{\infty }$ stabilize the system in *Ω*.*

## 3. Main Results

### 3.1. PI-Based Algorithm for Known Switched Systems

The offline, online and concurrent implementation of the aforementioned PI approach for switched systems with known dynamics has been investigated in references [21,22,38]. In this subsection, a novel online PI-based algorithm is proposed to approximate the optimal switching policy quickly.

Common online algorithms keep collecting data from the systems to evaluate and improve the switching policy. To be specific, once a new switching policy is produced at each iteration, it is applied to the system and the newly produced data is collected to calculate a new cost and a new switching policy. It entails considerable time to apply the new switching policy, collect new data and calculate a new cost and a new switching policy sequentially at each iteration. Aiming at this, inspired by the off-policy ADP methods for ordinary systems [36,39,40], an online PI-based algorithm is proposed to approximate the optimal switching policy quickly with only an initial switching policy applied and only the data produced by the initial switching policy is required. Next, the algorithm starts from the initial admissible switching policy.

With the initial admissible switching policy $v^{0}$ applied only, large amounts of data can be produced in the process and the state trajectory x(t) corresponding to the switching policy $v^{0}$ can be acquired which will be employed in the subsequent derivation. Along the acquired state trajectory x(t), $V^{k}\left(x\left(t+\delta t\right)\right)-V^{k}\left(x\left(t\right)\right)$ can be represented as

(9) $$V^{k}\left(x\left(t+\delta t\right)\right)-V^{k}\left(x\left(t\right)\right)=\int_{t}^{t+\delta t}{\left(\frac{\partial V^{k}\left(x\right)}{\partial x}\right)}^{T}f_{v^{0}\left(x\right)}\left(x\right)d\tau ,$$

where δt>0 is very small. To combine the policy iteration and the cost at the acquired state, integrating (7) along the acquired state trajectory x(t) and adding the integration to (9) yield

(10) $$V^{k}\left(x\left(t+\delta t\right)\right)-V^{k}\left(x\left(t\right)\right)=\int_{t}^{t+\delta t}{\left(\frac{\partial V^{k}\left(x\right)}{\partial x}\right)}^{T}\left(f_{v^{0}\left(x\right)}\left(x\right)-f_{v^{k}\left(x\right)}\left(x\right)\right)d\tau -\int_{t}^{t+\delta t}Q(x)d\tau .$$

The cost $V^{k}\left(x\right)$ is unknown. However, for all x∈Ω, it can be expressed by:

(11) $$V^{k}\left(x\right)=W^{k}\Phi \left(x\right)+e_{\Phi }^{k}\left(x\right),$$

where $\Phi \left(x\right)={\left[\Phi_{1}\left(x\right),\Phi_{2}\left(x\right),\ldots ,\Phi_{N_{w}}\left(x\right)\right]}^{T}$ is a vector concerning a set of linearly independent basis functions $\Phi_{j}\left(x\right):R^{n}\to R\left(j=1,2,\ldots ,N_{w}\right)$, $W^{k}\in R^{1\times N_{w}}$ is the weight vector and $e_{\Phi }^{k}\left(x\right)$ is the approximation error. $N_{w}$ is the number of the basis functions. A set of basis functions can constitute a particular basis of a function space and can almost approximate any function in the function space. With the approximation function (11) applied, Equation (10) can be transformed into

(12) $$W^{k}\left[\Phi \left(x\left(t+\delta t\right)\right)-\Phi \left(x\left(t\right)\right)+\int_{t}^{t+\delta t}\frac{\partial \Phi (x)}{\partial x}\left(f_{v^{k}\left(x\right)}\left(x\right)-f_{v^{0}\left(x\right)}\left(x\right)\right)d\tau \right]=-\int_{t}^{t+\delta t}Q(x)d\tau +e_{1}^{k}\left(t\right),$$

where $\frac{\partial (\cdot )}{\partial x}={\left[\frac{\partial {\left(\cdot \right)}_{1}}{\partial x},\frac{\partial {\left(\cdot \right)}_{2}}{\partial x},\ldots ,\frac{\partial {\left(\cdot \right)}_{m}}{\partial x}\right]}^{T}\in R^{m\times n}$ when $\left(\cdot \right)={\left[{\left(\cdot \right)}_{1},{\left(\cdot \right)}_{2},\ldots ,{\left(\cdot \right)}_{m}\right]}^{T}\in R^{m}$ and $e_{1}^{k}\left(t\right)=e_{\Phi }^{k}\left(x\left(t\right)\right)-e_{\Phi }^{k}\left(x\left(t+\delta t\right)\right)+\int_{t}^{t+\delta t}\frac{\partial e_{\Phi }^{k}\left(x\right)}{\partial x}\left(f_{v^{0}\left(x\right)}\left(x\right)-f_{v^{k}\left(x\right)}\left(x\right)\right)d\tau$ is the approximation error. Then the estimation can be achieved:

(13) $${\hat{W}}^{k}\left[\Phi \left(x\left(t+\delta t\right)\right)-\Phi \left(x\left(t\right)\right)+\int_{t}^{t+\delta t}\frac{\partial \Phi (x)}{\partial x}\left(f_{{\hat{v}}^{k}\left(x\right)}\left(x\right)-f_{v^{0}\left(x\right)}\left(x\right)\right)d\tau \right]=-\int_{t}^{t+\delta t}Q(x)d\tau ,$$

where the estimate ${\hat{v}}^{k}\left(x\right)$ is achieved through substituting the estimate ${\hat{W}}^{k}$ and the approximation (11) into (8) as follows:

(14) $${\hat{v}}^{k+1}\left(x\right)=\underset{i\in V}{arg\min}\left({\hat{W}}^{k}\frac{\partial \Phi (x)}{\partial x}f_{i}\left(x\right)\right),$$

with the initial switching policy estimate ${\hat{v}}^{0}\left(x\right)=v^{0}\left(x\right)$.

To employ the acquired state data corresponding to some selected instants, some data matrices can be defined as follows:

$$\bar{\Phi }\left(t_{r}\right)=\Phi \left(x\left(t_{r}+\delta t\right)\right)-\Phi \left(x\left(t_{r}\right)\right)$$

$$b\left(t_{r}\right)=\int_{t_{r}}^{t_{r}+\delta t}\frac{\partial \Phi (x)}{\partial x}\left(f_{{\hat{v}}^{k}\left(x\right)}\left(x\right)-f_{v^{0}\left(x\right)}\left(x\right)\right)d\tau$$

$$d\left(t_{r}\right)=\int_{t_{r}}^{t_{r}+\delta t}Q(x)d\tau$$

where $t_{1}<t_{2}<\cdots <t_{l}$ are the selected instants, *l* is a positive integer, r=1,2,…l, $\bar{\Phi }\left(t_{r}\right)\in R^{N_{w}}$, $b\left(t_{r}\right)\in R^{N_{w}}$ and $d(t_{r})\in R$. The following assumption concerning the data matrices is made as [40,41]:

**Assumption** **2.** *There exist a positive integer $\bar{L}$ and a positive number α such that for all $L\geq \bar{L}$, the following equality holds:*

$$\frac{1}{L}\sum_{r=1}^{L}(\bar{\Phi }\left(t_{r}\right)+b\left(t_{r}\right)){\left(\bar{\Phi }\left(t_{r}\right)+b\left(t_{r}\right)\right)}^{T}\geq \alpha I_{N_{w}}.$$

In optimal control of ordinary systems, this kind of assumption can be satisfied by exerting an exploration noise in the input [39,40]. In the case of the switched systems, according to [21,22], it can be satisfied through random switching.

Then based on Assumption 2, ${\hat{W}}^{k}$ can be achieved with the data matrices as the following formula:

(15) $${\hat{W}}^{k}=-\left[\sum_{r=1}^{L}d\left(t_{r}\right){\left(\bar{\Phi }\left(t_{r}\right)+b\left(t_{r}\right)\right)}^{T}\right]\cdot {\left[\sum_{r=1}^{L}(\bar{\Phi }\left(t_{r}\right)+b\left(t_{r}\right)){\left(\bar{\Phi }\left(t_{r}\right)+b\left(t_{r}\right)\right)}^{T}\right]}^{-1}.$$

According to the analysis, the online PI-based algorithm for switched systems with known dynamics can be formulated in Algorithm 1.

**Algorithm 1** Online policy iteration (PI)-based Algorithm.Step 1. Start with an initial admissible switching policy $v^{0}\left(x\right)$ and set the iteration index k=0. Step 2. Apply $v^{0}\left(x\right)$ in the switched systems and acquire the state data. Set ${\hat{v}}^{0}\left(x\right)=v^{0}\left(x\right)$. Calculate $\bar{\Phi }\left(t_{r}\right)$ and $d(t_{r})$ for r=1,2,…,l according to their definition with the state data. Step 3. Calculate $b(t_{r})$ for r=1,2,…,l according to its definition with the state data and then calculate ${\hat{W}}^{k}$ from (15). Step 4. Update the switching policy ${\hat{v}}^{k+1}\left(x\right)$ as (14). Step 5. If $∥{\hat{W}}_{}^{k}-{\hat{W}}_{}^{k-1}∥<\varepsilon$, set ${\hat{W}}_{}^{*}={\hat{W}}_{}^{k}$ and exit. Otherwise, set k=k+1 and go back to Step 3.

In the algorithm, when the switching policy $v^{0}\left(x\right)$ is applied, the corresponding system state data can be obtained. With multiple samples, multiple data matrix $\bar{\Phi }\left(t_{r}\right)$ and $b(t_{r})$ will be calculated. Sufficient samples should be employed to satisfy that $\frac{1}{L}\sum_{r=1}^{L}(\bar{\Phi }\left(t_{r}\right)+b\left(t_{r}\right)){\left(\bar{\Phi }\left(t_{r}\right)+b\left(t_{r}\right)\right)}^{T}\geq \alpha I_{N_{w}}$ with a proper α so that the sampling stage must be long enough to ensure that. Moreover, since the weight vector ${\hat{W}}^{k}$ to be solved in Equation (15) has $N_{w}$ components, at least $N_{w}$ instants $t_{r}$ should be employed to solve ${\hat{W}}^{k}$ so that *r* is no less than $N_{w}$. Then through the algorithm, the approximate optimal cost function can be achieved as ${\hat{V}}^{*}\left(x\right)={\hat{W}}^{*}\Phi \left(x\right)$ with the corresponding approximate optimal switching policy ${\hat{v}}^{*}\left(x\right)=\underset{i\in V}{arg\min}\left({\hat{W}}^{*}\frac{\partial \Phi (x)}{\partial x}f_{i}\left(x\right)\right)$. It can be seen from Algorithm 1 that only the initial switching policy is applied and then Algorithm 1 can approximate the optimal switching policy quickly with the data produced by the initial switching policy.

### 3.2. Data-Driven PI-Based Algorithm for Unknown Switched Systems

In this subsection, based on the proposed PI approach dependent on system models, a data-driven PI-based algorithm is proposed for switched systems with unknown subsystems. The algorithm takes full advantage of data produced by an initial switching policy to approximate the optimal switching policy quickly.

From Section 3.1, with the initial admissible switching policy $v^{0}$ applied and along the acquired state trajectory x(t), (10) can be achieved and the cost function can be represented by (11). In the process, $\frac{\partial V^{k}\left(x\right)}{\partial x}$ can be represented by ${\left(W^{k}\frac{\partial \Phi (x)}{\partial x}\right)}^{T}$ and solved with known system models. However, due to the unknown subsystem models, $W^{k}\frac{\partial \Phi (x)}{\partial x}f_{i}\left(x\right)$ representing ${\left(\frac{\partial V^{k}\left(x\right)}{\partial x}\right)}^{T}f_{i}\left(x\right)$ cannot work well as Section 3.1. So another approximation function is brought in to solve the problem. For all x∈Ω, the unknown variable ${\left(\frac{\partial V^{k}\left(x\right)}{\partial x}\right)}^{T}f_{i}\left(x\right),i\in V$ can be expressed by:

(16) $${\left(\frac{\partial V^{k}\left(x\right)}{\partial x}\right)}^{T}f_{i}\left(x\right)=C_{i}^{k}\Psi \left(x\right)+e_{\Psi ,i}^{k}\left(x\right),$$

where $\Psi \left(x\right)={\left[\Psi_{1}\left(x\right),\Psi_{2}\left(x\right),\ldots ,\Psi_{N_{c}}\left(x\right)\right]}^{T}$ is a vector concerning a set of linearly independent basis functions $\Psi_{j}\left(x\right):R^{n}\to R\left(j=1,2,\ldots ,N_{c}\right)$, $C_{i}^{k},i\in V$ are the weight vectors and $e_{\Psi ,i}^{k}\left(x\right)$ is the approximation error. $N_{c}$ is the number of the basis functions.

With the approximation functions (11) and (16) applied, Equation (10) can be transformed into

(17) $$W^{k}\left(\Phi \left(x\left(t+\delta t\right)\right)-\Phi \left(x\left(t\right)\right)\right)+\int_{t}^{t+\delta t}\left(C_{v^{k}\left(x\right)}^{k}-C_{v^{0}\left(x\right)}^{k}\right)\Psi \left(x\right)d\tau =-\int_{t}^{t+\delta t}Q(x)d\tau +e_{2}^{k}\left(t\right),$$

where $e_{2}^{k}\left(t\right)=\int_{t}^{t+\delta t}\left(e_{\Psi ,v^{0}\left(x\right)}^{k}\left(x\right)-e_{\Psi ,v^{k}\left(x\right)}^{k}\left(x\right)\right)d\tau +e_{\Psi }^{k}\left(x\left(t\right)\right)-e_{\Psi }^{k}\left(x\left(t+\delta t\right)\right)$ is the approximation error. Since δt is very small, the values of $v^{k}\left(x\right)$ and $v^{0}\left(x\right)$ can be seen to be constant during the time interval [t,t+δt) so that $C_{v^{0}\left(x\right)}^{k}$ and $C_{v^{k}\left(x\right)}^{k}$ are constant during the time interval [t,t+δt). Therefore, the estimation can be made as (18):

(18) $${\hat{W}}^{k}\left(\Phi \left(x\left(t+\delta t\right)\right)-\Phi \left(x\left(t\right)\right)\right)+\left({\hat{C}}_{{\hat{v}}^{k}\left(x\right)}^{k}-{\hat{C}}_{v^{0}\left(x\right)}^{k}\right)\int_{t}^{t+\delta t}\Psi (x)d\tau =-\int_{t}^{t+\delta t}Q(x)d\tau .$$

For the subsequent algorithm, in addition to the data matrices defined in Section 3.1, some more matrices are required which are defined as follows:

$$g\left(t_{r}\right)=\int_{t_{r}}^{t_{r}+\delta t}\Psi (x(\tau ))d\tau$$

where $t_{1}<t_{2}<\cdots <t_{l}$, *l* is a positive integer, $g\left(t_{r}\right)\in R^{N_{c}}$ and r=1,2,…l.

At first, the following assumption concerning the data matrices is made as Assumption 2:

**Assumption** **3.** *There exist a positive integer ${\bar{L}}^{\prime }$ and a positive number α such that for all $L\geq {\bar{L}}^{\prime }$, the following equalities hold:*

$$\frac{1}{L}\sum_{r=1}^{L}\bar{\Phi }\left(t_{r}\right)\bar{\Phi }{\left(t_{r}\right)}^{T}\geq \alpha I_{N_{w}}$$

*where the time instants $t_{r}$ satisfies the condition that ${\hat{v}}^{k}\left(x\left(t_{r}\right)\right)=v^{0}\left(x\left(t_{r}\right)\right)$;*

$$\frac{1}{L}\sum_{r=1}^{L}\Psi \left(x\left(t_{r}\right)\right)\Psi {\left(x\left(t_{r}\right)\right)}^{T}\geq \alpha I_{N_{c}}$$

*where the time instants $t_{r}$ satisfies the condition that $v^{0}\left(x\left(t_{r}\right)\right)=i$ for ∀i∈V;*

$$\frac{1}{L}\sum_{1}^{L}g\left(t_{r}\right)g{\left(t_{r}\right)}^{T}\geq \alpha I_{N_{c}}$$

*where the time instants $t_{r}$ satisfies the conditions that ${\hat{v}}^{k}\left(x\left(t_{r}\right)\right)=i$ and $v^{0}\left(x\left(t_{r}\right)\right)=j$ for ∀i≠j,(i,j∈V).*

**Remark** **1.** *Since the vector ${\hat{C}}_{{\hat{v}}^{k}\left(x\right)}^{k}-{\hat{C}}_{v^{0}\left(x\right)}^{k}$ changes as x changes, the weight vector ${\hat{W}}^{k}$ and ${\hat{C}}_{{\hat{v}}^{k}\left(x\right)}^{k}$ cannot be solved directly from (18) with the data matrices as Section 3.1. The difficulty in this problem is mainly calculating the weight vector ${\hat{W}}^{k}$ and ${\hat{C}}_{{\hat{v}}^{k}\left(x\right)}^{k}$ or achieve enough useful knowledge about the weight vectors through the state data.*

Based on the above analysis, the following approach is designed to acquire useful knowledge about the weight vector ${\hat{W}}^{k}$ and ${\hat{C}}_{{\hat{v}}^{k}\left(x\right)}^{k}$ step by step through different state data. The weight vector $W^{k}$ is discussed firstly. The state data satisfying the condition that ${\hat{v}}^{k}\left(x\right)=v^{0}\left(x\right)$ is selected from all the state data and then utilized in (18). It is obvious that when ${\hat{v}}^{k}\left(x\right)=v^{0}\left(x\right)$, ${\hat{C}}_{{\hat{v}}^{k}\left(x\right)}^{k}={\hat{C}}_{v^{0}\left(x\right)}^{k}$ and (18) can be simplified to:

(19) $${\hat{W}}^{k}\left(\Phi \left(x\left(t+\delta t\right)\right)-\Phi \left(x\left(t\right)\right)\right)=-\int_{t}^{t+\delta t}Q(x)d\tau .$$

Under Assumption 3, the estimate of the weight vector $W^{k}$ can be achieved from (19) with the data matrices defined in Section 3.1 concerning certain states as follows:

(20) $${\hat{W}}^{k}=-(\sum_{r=1}^{L}d\left(t_{r}\right)\bar{\Phi }{\left(t_{r}\right)}^{T}){\left(\sum_{r=1}^{L}\bar{\Phi }\left(t_{r}\right)\bar{\Phi }{\left(t_{r}\right)}^{T}\right)}^{-1},$$

where the time instants $t_{r}$ satisfies the condition that ${\hat{v}}^{k}\left(x\left(t_{r}\right)\right)=v^{0}\left(x\left(t_{r}\right)\right)$.

Then, we concentrate on estimating the weight vector of $C_{i}^{k}$. Along the acquired state trajectory x(t) produced by the switching policy $v^{0}$, it can be obtained from (16) and (7) that when k=0 the following formula holds:

(21) $$\int_{t}^{t+\delta t}C_{v^{0}\left(x\right)}^{0}\Psi \left(x\right)d\tau +\int_{t}^{t+\delta t}e_{\Psi ,v^{0}\left(x\right)}^{0}\left(x\right)d\tau +\int_{t}^{t+\delta t}Q(x)d\tau =0.$$

The estimation can be made as follows:

$${\hat{C}}_{v^{0}\left(x\right)}^{0}\int_{t}^{t+\delta t}\Psi (x)d\tau +\int_{t}^{t+\delta t}Q(x)d\tau =0.$$

Under Assumption 3, the estimate of the weight vector $C_{i}^{0}$ can be easily achieved as follows:

(22) $${\hat{C}}_{i}^{0}=-[\sum_{1}^{L}d\left(t_{r}\right)g{\left(t_{r}\right)}^{T}]{\left(\sum_{1}^{L}g\left(t_{r}\right)g{\left(t_{r}\right)}^{T}\right)}^{-1},$$

where the time instants $t_{r}$ satisfies the condition that $v^{0}\left(x\left(t_{r}\right)\right)=i\left(i\in V\right)$.

Though the weight vector $C_{i}^{k}$ is not easy to estimate, the estimate of $C_{i}^{k}-C_{j}^{k}$(i≠j) can be easily achieved from (18) with the data matrices concerning certain states under Assumption 3 as follows:

(23) $${\hat{C}}_{i}^{k}-{\hat{C}}_{j}^{k}=-[\sum_{1}^{L}\left(d\left(t_{r}\right)+{\hat{W}}^{k}\bar{\Phi }\left(t_{r}\right)\right)g{\left(t_{r}\right)}^{T}]{\left(\sum_{1}^{L}g\left(t_{r}\right)g{\left(t_{r}\right)}^{T}\right)}^{-1},$$

where the time instants $t_{r}$ satisfies the conditions that ${\hat{v}}^{k}\left(x\left(t_{r}\right)\right)=i$ and $v^{0}\left(x\left(t_{r}\right)\right)=j$(i,j∈V and i≠j).

For a certain state *x*, ${\left(\frac{\partial V^{k}\left(x\right)}{\partial x}\right)}^{T}f_{v^{0}\left(x\right)}\left(x\right)$ is constant and $\underset{i\in V}{arg\min}\left({\left(\frac{\partial V^{k}\left(x\right)}{\partial x}\right)}^{T}f_{i}\left(x\right)\right)=\underset{i\in V}{arg\min}\left({\left(\frac{\partial V^{k}\left(x\right)}{\partial x}\right)}^{T}f_{i}\left(x\right)-{\left(\frac{\partial V^{k}\left(x\right)}{\partial x}\right)}^{T}f_{v^{0}\left(x\right)}\left(x\right)\right)$. Therefore, on the basis of (8), employing the estimate of (22) and (23), the switching policy $v^{k+1}\left(x\right)$ is estimated by

(24) $${\hat{v}}^{k+1}\left(x\right)=\left\{\begin{array}{cc} \underset{i\in V}{arg\min}\left({\hat{C}}_{i}^{0}\Psi \left(x\right)\right) & k=0\\ \underset{i\in V}{arg\min}\left({\hat{C}}_{i}^{k}-{\hat{C}}_{v^{0}\left(x\right)}^{k}\right)\Psi \left(x\right) & k>0 \end{array}\right.$$

with the initial switching policy estimate ${\hat{v}}^{0}\left(x\right)=v^{0}\left(x\right)$.

The approach utilizes different parts of the acquired state data to calculate the weight vector ${\hat{W}}^{k}$, $C_{i}^{0}$ and $C_{i}^{k}-C_{j}^{k}$(i≠j) respectively. Then the weight vectors are employed to calculate the switching policy and the cost function.

According to the aforementioned analysis, the data-driven PI-based algorithm for switched systems can be formulated as follows:

Then, the approximate optimal cost function can be achieved as ${\hat{V}}^{*}\left(x\right)={\hat{W}}^{*}\Phi \left(x\right)$ with the corresponding approximate optimal switching policy ${\hat{v}}^{*}\left(x\right)=\underset{i\in V}{arg\min}\left(\left({\hat{C}}_{i}^{k}-{\hat{C}}_{v^{0}\left(x\right)}^{k}\right)\Psi \left(x\right)\right)$.

**Remark** **2.** *In Algorithm 2, only the initial switching policy $v^{0}\left(x\right)$ requires to be applied in the system and the produced state data is collected at the beginning. The data matrices $\bar{\Phi }\left(t_{r}\right)$, $d(t_{r})$ and $g(t_{r})$ are calculated once at the beginning and don’t require to be calculated repeatedly at each iteration. It is very convenient and timesaving to operate online according to Algorithm 2 and the calculation time is very short. Therefore, the optimal cost can be approximated rapidly in Algorithm 2. Moreover, Algorithm 2 is only based on data with no need for the knowledge of subsystems.*

**Algorithm 2** Data-driven PI-based algorithm.Step 1. Start with an initial admissible switching policy $v^{0}\left(x\right)$ and set the iteration index k=0. Step 2. Apply $v^{0}\left(x\right)$ in the switched systems and acquire the state data. Set ${\hat{v}}^{0}\left(x\right)=v^{0}\left(x\right)$. Calculate $\bar{\Phi }\left(t_{r}\right)$, $d(t_{r})$ and $g(t_{r})$ for r=1,2,…,l according to their definition with the state data. Step 3. Calculate ${\hat{C}}_{i}^{0}$ for all i∈V from (22) and then calculate ${\hat{v}}^{1}\left(x\right)$ from (24). Set k=1. Step 4. Calculate ${\hat{W}}^{k}$ from (20) and then calculate ${\hat{C}}_{i}^{k}-{\hat{C}}_{j}^{k}$ from (23) for all i≠j(i,j∈V). Step 5. Update the switching policy ${\hat{v}}^{k+1}\left(x\right)$ as (24). Step 6. If $∥{\hat{W}}_{}^{k}-{\hat{W}}_{}^{k-1}∥<\varepsilon$, set ${\hat{W}}_{}^{*}={\hat{W}}_{}^{k}$ and exit. Otherwise, set k=k+1 and go back to Step 4.

Next, the convergence of Algorithm 2 is analyzed. Based on Theorem 1, Theorem 2 is stated for the convergence analysis of Algorithm 2.

**Theorem** **2.** *For system (1) with cost (2) under Assumptions 1 and 3, if the value function sequence ${\left\{V^{k}\right\}}_{k=0}^{\infty }$ and the switching policy sequence ${\left\{v^{k}\right\}}_{k=0}^{\infty }$ are generated through (7) and (8) initiating from an initial admissible policy $v^{0}$, for ∀ε>0 and all x∈Ω, there exists $\bar{N}>0$ such that for $\forall N>\bar{N}$, when δt approaches to zero, the following inequalities hold:*

$$\left|{\hat{V}}^{k}\left(x\right)-V^{k}\left(x\right)\right|<\varepsilon ,{\hat{v}}^{k}\left(x\right)=v^{k}\left(x\right),$$

*where $N=\min\{N_{w},N_{c}\}$, ${\hat{V}}^{k}\left(x\right)={\hat{W}}^{k}\Phi \left(x\right)$, ${\hat{W}}^{k}$ and ${\hat{v}}^{k}$ are generated through (20) and (24) in Algorithm 2, and k=0,1,….*

**Proof of Theorem** **2.** Mathematical induction is utilized to prove the convergence.

Firstly, we discuss the theorem when k=0. When k=0, ${\hat{v}}^{0}\left(x\right)=v^{0}\left(x\right)$, ${\hat{C}}_{{\hat{v}}^{0}\left(x\right)}^{k}={\hat{C}}_{v^{0}\left(x\right)}^{k}$ and it can be achieved from (17) and (18) that:

$$\left({\hat{W}}^{0}-W^{0}\right)\left(\Phi \left(x\left(t+\delta t\right)\right)-\Phi \left(x\left(t\right)\right)\right)=-e_{2}^{0}\left(t\right)$$

and

$$\sum_{r=1}^{L}\left({\hat{W}}^{0}-W^{0}\right)\bar{\Phi }\left(t_{r}\right){\left(\bar{\Phi }\left(t_{r}\right)\right)}^{T}{\left({\hat{W}}^{0}-W^{0}\right)}^{T}=\sum_{r=1}^{L}e_{2}^{0}{\left(t_{r}\right)}^{2}.$$

According to Assumption 3, it follows that

$$\frac{1}{L}\sum_{r=1}^{L}\bar{\Phi }\left(t_{r}\right){\left(\bar{\Phi }\left(t_{r}\right)\right)}^{T}\geq \alpha I_{N_{w}}$$

and then ${∥{\hat{W}}^{0}-W^{0}∥}^{2}\leq \frac{1}{\alpha L}\sum_{r=1}^{L}e_{2}^{0}{\left(t_{r}\right)}^{2}$. The approximation theory [42] yields that for all x∈Ω, $\lim_{N_{w}\to \infty }e_{\Phi }^{0}\left(x\right)=0$, then $\lim_{N_{w}\to \infty }e_{2}^{0}\left(t_{r}\right)=0$. Therefore, for ∀ε>0 and ∀x∈Ω, there exists ${\bar{N}}_{w}>0$ such that for $\forall N_{w}>{\bar{N}}_{w}$, ${∥{\hat{W}}^{0}-W^{0}∥}^{2}\leq \varepsilon$ and then $\left|{\hat{V}}^{0}\left(x\right)-V^{0}\left(x\right)\right|<\varepsilon$. It follows that $\lim_{N_{w}\to \infty }∥{\hat{W}}^{0}-W^{0}∥=0$ and $\lim_{N_{w}\to \infty }{\hat{V}}^{0}\left(x\right)=V^{0}\left(x\right)$ for ∀x∈Ω. Then, it can be achieved from (21) and (22) that:

$$\left({\hat{C}}_{v^{0}\left(x\right)}^{0}-C_{v^{0}\left(x\right)}^{0}\right)\int_{t}^{t+\delta t}\Psi (x)d\tau =\int_{t}^{t+\delta t}e_{\Psi ,v^{0}\left(x\right)}^{0}\left(x\right)d\tau +\int_{t}^{t+\delta t}\left(C_{v^{0}\left(x\left(\tau \right)\right)}^{0}-C_{v^{0}\left(x\left(t\right)\right)}^{0}\right)\Psi \left(x\right)d\tau .$$

Similarly, the approximation theory [42] yields that for all x∈Ω, $\lim_{N_{w}\to \infty }e_{\Psi ,i}^{0}\left(x\right)=0\left(i\in V\right)$ and then $\lim_{\delta t\to 0}\int_{t}^{t+\delta t}\left(C_{v^{0}\left(x\left(\tau \right)\right)}^{0}-C_{v^{0}\left(x\left(t\right)\right)}^{0}\right)\Psi \left(x\right)d\tau =0$. Under Assumption 3, it can be deduced that for ∀x∈Ω, limNc→∞δt→0(∂V0(x)∂x)^Tfi(x)=(∂V0(x)∂x)Tfi(x). It implies that limNc→∞δt→0v^1(x)=v1(x) for ∀x∈Ω.

Secondly, we consider the theorem when k=1. When k=1, $\lim_{N_{c}\to \infty }{\hat{C}}_{{\hat{v}}^{1}\left(x\right)}^{1}={\hat{C}}_{v^{1}\left(x\right)}^{1}$. When ${\hat{W}}^{1}$ is calculated, for ∀x∈Ω and it can be achieved from (17) to (19) that:

$$\begin{array}{c} \left({\hat{W}}^{1}-W^{1}\right)\left(\Phi \left(x\left(t+\delta t\right)\right)-\Phi \left(x\left(t\right)\right)\right)+\left({\hat{C}}_{{\hat{v}}^{1}\left(x\right)}^{1}-{\hat{C}}_{v^{0}\left(x\right)}^{1}\right)\int_{t}^{t+\delta t}\Psi (x)d\tau -\\ \int_{t}^{t+\delta t}\left(C_{v^{1}\left(x\right)}^{1}-C_{v^{0}\left(x\right)}^{1}\right)\Psi \left(x\right)d\tau =-e_{2}^{1}\left(t\right), \end{array}$$

where ${\hat{v}}^{1}\left(x\right)=v^{0}\left(x\right)$ and thus $\lim_{N_{c}\to \infty }v^{1}\left(x\right)=v^{0}\left(x\right)$ for ∀x∈Ω. Then it can be inferred that when $N_{c}\to \infty$

$$\left({\hat{W}}^{1}-W^{1}\right)\left(\Phi \left(x\left(t+\delta t\right)\right)-\Phi \left(x\left(t\right)\right)\right)=-e_{2}^{1}\left(t\right)$$

Under Assumption 3, it can be deduced that for all x∈Ω, $\lim_{N_{w}\to \infty }e_{2}^{1}\left(t_{r}\right)=0$, $\lim_{N_{w},N_{c}\to \infty }∥{\hat{W}}^{1}-W^{1}∥=0$ and $\lim_{N_{w},N_{c}\to \infty }{\hat{V}}^{1}\left(x\right)=V^{1}\left(x\right)$. Then, it can be achieved from (17), (18) and (23) that:

$$\begin{array}{c} \;\;\;\;\left({\hat{C}}_{{\hat{v}}^{1}\left(x\right)}^{1}-{\hat{C}}_{v^{0}\left(x\right)}^{1}\right)\int_{t}^{t+\delta t}\Psi (x)d\tau -\int_{t}^{t+\delta t}\left(C_{v^{1}\left(x\right)}^{1}-C_{v^{0}\left(x\right)}^{1}\right)\Psi \left(x\right)d\tau \\ =-e_{2}^{1}\left(t\right)-\left({\hat{W}}^{1}-W^{1}\right)\left(\Phi \left(x\left(t+\delta t\right)\right)-\Phi \left(x\left(t\right)\right)\right). \end{array}$$

Since it can be achieved that $\int_{t}^{t+\delta t}\left(C_{v^{1}\left(x\right)}^{1}-C_{v^{0}\left(x\right)}^{1}\right)\Psi \left(x\right)d\tau =\left(C_{v^{1}\left(x\right)}^{1}-C_{v^{0}\left(x\right)}^{1}\right)\int_{t}^{t+\delta t}\Psi (x)d\tau +e_{3}^{1}\left(t\right)$ where $e_{3}^{1}\left(t\right)=\int_{t}^{t+\delta t}(\left(C_{v^{1}\left(x\left(\tau \right)\right)}^{1}-C_{v^{1}\left(x\left(t\right)\right)}^{1}\right)-\left(C_{v^{0}\left(x\left(\tau \right)\right)}^{1}-C_{v^{0}\left(x\left(t\right)\right)}^{1}\right)\Psi \left(x\right)d\tau$, it can be inferred that

$$\begin{array}{c} \;\;\;\left[\left({\hat{C}}_{{\hat{v}}^{1}\left(x\right)}^{1}-{\hat{C}}_{v^{0}\left(x\right)}^{1}\right)-\left(C_{v^{1}\left(x\right)}^{1}-C_{v^{0}\left(x\right)}^{1}\right)\right]\int_{t}^{t+\delta t}\Psi (x)d\tau \\ =e_{3}^{1}\left(t\right)-e_{2}^{1}\left(t\right)-\left({\hat{W}}^{1}-W^{1}\right)\left(\Phi \left(x\left(t+\delta t\right)\right)-\Phi \left(x\left(t\right)\right)\right). \end{array}$$

Since $\lim_{\delta t\to 0}e_{3}^{1}\left(t\right)=0$, $\lim_{N_{w}\to \infty }e_{2}^{1}\left(t\right)=0$ and $\lim_{N_{w},N_{c}\to \infty }∥{\hat{W}}^{1}-W^{1}∥=0$, under Assumption 3, it can be deduced that limNw,Nc→∞δt→0(C^v^1(x)1−C^v0(x)1)−(Cv1(x)1−Cv0(x)1)=0 and then limNw,Nc→∞δt→0(C^v^1(x)1−C^v0(x)1)Ψ(x)=(Cv1(x)1−Cv0(x)1)Ψ(x) for ∀x∈Ω. It implies that limNw,Nc→∞δt→0v^2(x)=v2(x) for ∀x∈Ω.

Thirdly, we suppose the theorem holds when k=k. That is to say, limNw,Nc→∞δt→0V^k(x)=Vk(x) and limNw,Nc→∞δt→0v^k+1(x)=vk+1(x) for ∀x∈Ω. When ${\hat{W}}^{k+1}$ is calculated, it can be achieved from (17) to (19) that:

$$\begin{array}{c} \left({\hat{W}}^{k+1}-W^{k+1}\right)\left(\Phi \left(x\left(t+\delta t\right)\right)-\Phi \left(x\left(t\right)\right)\right)+\left({\hat{C}}_{{\hat{v}}^{k+1}\left(x\right)}^{k+1}-{\hat{C}}_{v^{0}\left(x\right)}^{k+1}\right)\int_{t}^{t+\delta t}\Psi (x)d\tau -\\ \int_{t}^{t+\delta t}\left(C_{v^{k+1}\left(x\right)}^{k+1}-C_{v^{0}\left(x\right)}^{k+1}\right)\Psi \left(x\right)d\tau =-e_{2}^{k+1}\left(t\right) \end{array}$$

where ${\hat{v}}^{k+1}\left(x\right)=v^{0}\left(x\right)$ and limNw,Nc→∞δt→0vk+1(x)=v0(x) for ∀x∈Ω. Then it can be inferred that

$$\left({\hat{W}}^{k+1}-W^{k+1}\right)\left(\Phi \left(x\left(t+\delta t\right)\right)-\Phi \left(x\left(t\right)\right)\right)=-e_{2}^{k+1}\left(t\right)$$

Under Assumption 3, it can be deduced that limNw,Nc→∞δt→0W^k+1−Wk+1=0 and limNw,Nc→∞δt→0V^k+1(x)=Vk+1(x) for ∀x∈Ω. Then, it can be achieved from (17), (18) and (23) that:

$$\begin{array}{c} \;\;\;\;\left[\left({\hat{C}}_{{\hat{v}}^{k+1}\left(x\right)}^{k+1}-{\hat{C}}_{v^{0}\left(x\right)}^{k+1}\right)-\left(C_{v^{k+1}\left(x\right)}^{k+1}-C_{v^{0}\left(x\right)}^{k+1}\right)\right]\int_{t}^{t+\delta t}\Psi (x)d\tau \\ =-\left({\hat{W}}^{k+1}-W^{k+1}\right)\left(\Phi \left(x\left(t+\delta t\right)\right)-\Phi \left(x\left(t\right)\right)\right)-e_{2}^{k+1}\left(t\right)+e_{3}^{k+1}\left(t\right), \end{array}$$

where $e_{3}^{k+1}\left(t\right)=\int_{t}^{t+\delta t}(\left(C_{v^{k+1}\left(x\left(\tau \right)\right)}^{k+1}-C_{v^{k+1}\left(x\left(t\right)\right)}^{k+1}\right)-\left(C_{v^{0}\left(x\left(\tau \right)\right)}^{k+1}-C_{v^{0}\left(x\left(t\right)\right)}^{k+1}\right)\Psi \left(x\right)d\tau$. Since $\lim_{\delta t\to 0}e_{3}^{k+1}\left(t\right)=0$, $\lim_{N_{w}\to \infty }e_{2}^{k+1}\left(t\right)=0$ and limNw,Nc→∞δt→0W^k+1−Wk+1=0, under Assumption 3, it can be deduced that limNw,Nc→∞δt→0(C^v^k+1(x)k+1−C^v0(x)k+1)−(Cvk+1(x)k+1−Cv0(x)k+1)=0 and then limNw,Nc→∞δt→0(C^v^k+1(x)k+1−C^v0(x)k+1)Ψ(x)=(Cvk+1(x)k+1−Cv0(x)k+1)Ψ(x) for ∀x∈Ω. It implies that limNw,Nc→∞δt→0v^k+2(x)=vk+2(x) for ∀x∈Ω. In brief, it can be deduced that the theorem holds when k=k+1 from the supposition that the theorem holds when k=k.

The proof is completed through mathematical induction. □

It can be achieved from Theorem 2 that the value function ${\hat{V}}^{k}\left(x\right)$ generated through Algorithm 2 is an approximation of $V^{k}\left(x\right)$ and the corresponding switching policy is ${\hat{v}}^{k}\left(x\right)=v^{k}\left(x\right)$ if the preconditions are satisfied. Theorem 2 combined with Theorem 1 indicates that the value function ${\hat{V}}^{*}\left(x\right)={\hat{W}}^{*}\Phi \left(x\right)$ is the approximate optimal cost function with the corresponding approximate optimal switching policy ${\hat{v}}^{*}\left(x\right)=\underset{i\in V}{arg\min}\left(\left({\hat{C}}_{i}^{k}-{\hat{C}}_{v^{0}\left(x\right)}^{k}\right)\Psi \left(x\right)\right)$.

**Remark** **3.** *In practice, the error of the approximation exists and the small parameter δt can not get infinitely close to zero so that the solution calculated from Algorithm 2 is suboptimal.*

## 4. Example

In this section, two examples are illustrated to validate the effectiveness of the suboptimal scheduling approach of Algorithm 1 and the data-driven suboptimal scheduling approach of Algorithm 2 in this paper.

**Example** **1.** *Consider a switched system as [21,38] consisting of the following subsystems:*

$$\dot{x}\left(t\right)=f_{1}\left(x\left(t\right)\right)=-x\left(t\right),\dot{x}\left(t\right)=f_{2}\left(x\left(t\right)\right)=-x^{3}\left(t\right)$$

*with x(0)=2 and $Q\left(x\left(t\right)\right)=x^{2}\left(t\right)$.*

The optimal switching policy can be known from [17] as

$$v^{*}\left(x\right)=\left\{\begin{array}{cc} 1 & x\leq 1\\ 2 & x>1 \end{array}\right.$$

Choose the initial switching policy $v^{0}\left(x\right)=1$ when x≤1.5 and $v^{0}\left(x\right)=2$ when x>1.5. The basis functions are $\Phi \left(x\right)={\left[x^{2},x^{4},x^{6},x^{8},x^{10}\right]}^{T}$ selected as [21]. Set the sample period δt=0.002 s.

**Figure 1:**
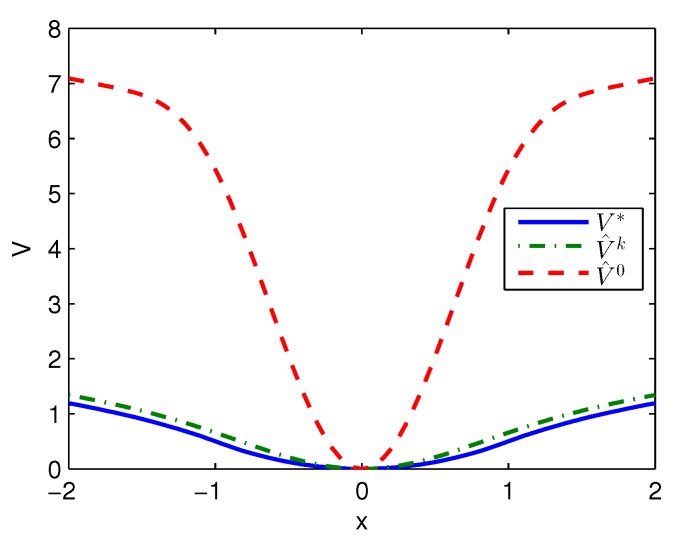
Costs achieved by Algorithm 1 applied in Example 1.

**Figure 2:**
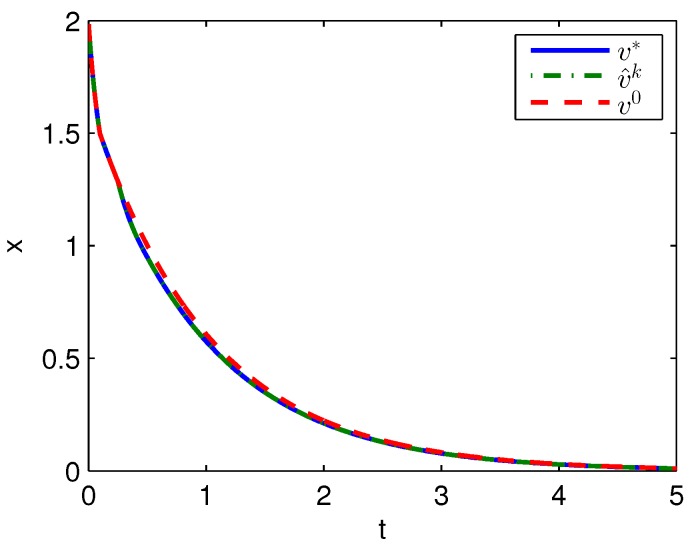
State trajectories with Algorithm 1 applied in Example 1.

**Figure 3:**
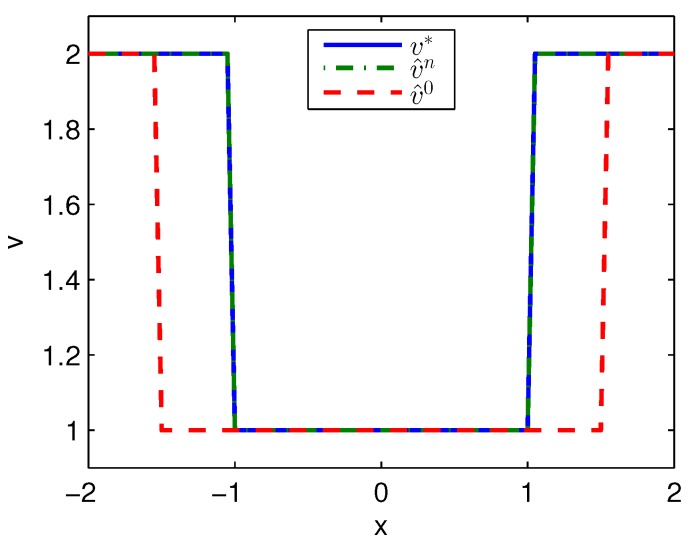
Switching policies achieved by Algorithm 1 applied in Example 1.

Apply Algorithm 1 and utilize the online state data from t=0 to 0.2 s. Then after calculation in 0.02 s, the approximate optimal cost is achieved with the corresponding approximate optimal switching policy through 3 iterations. The initial cost ${\hat{V}}^{0}$, the approximate optimal cost ${\hat{V}}^{k}$ and the optimal cost $V^{*}$ are demonstrated in Figure 1. The largest error between ${\hat{V}}^{k}$ and $V^{*}$ is 0.1621 and it is obvious that the approximate optimal cost ${\hat{V}}^{k}$ is very close to the optimal cost of $V^{*}$. The state trajectories with the initial switching policy $v^{0}$, the approximate optimal switching policy ${\hat{v}}^{k}$ and the optimal switching policy $v^{*}$ applied in the system after t=0.25 s are demonstrated in Figure 2, where the state trajectory corresponding to ${\hat{v}}^{k}$ coincides with the one corresponding to $v^{*}$ and their largest error is zero while the largest error between the state trajectory corresponding to $v^{0}$ and the one corresponding to $v^{*}$ is 0.0563. The online trajectories are too close to show the superiority of the proposed algorithm so that the switching policies $v^{0}$, ${\hat{v}}^{k}$ and $v^{*}$ when x∈[−2,2] are illustrated in Figure 3. Apparently, ${\hat{v}}^{k}$ and $v^{*}$ are the same. The similarity rate of the schedules ${\hat{v}}^{k}$ and $v^{*}$ is 100% while the similarity rate of the schedules $v^{0}$ and $v^{*}$ is 75.31%.

**Figure 4:**
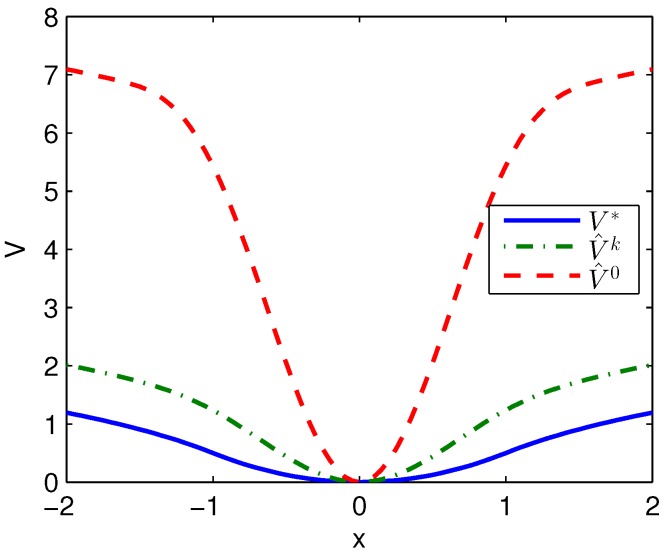
Costs achieved by Algorithm 2 applied in Example 1.

**Figure 5:**
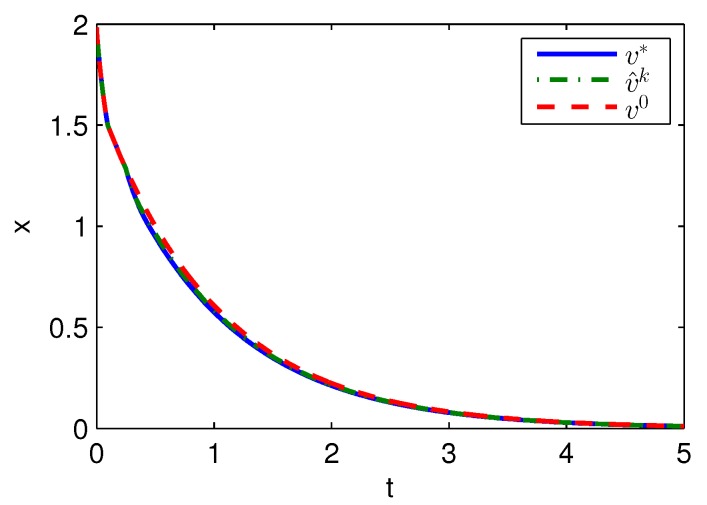
State trajectories with Algorithm 2 applied in Example 1.

**Figure 6:**
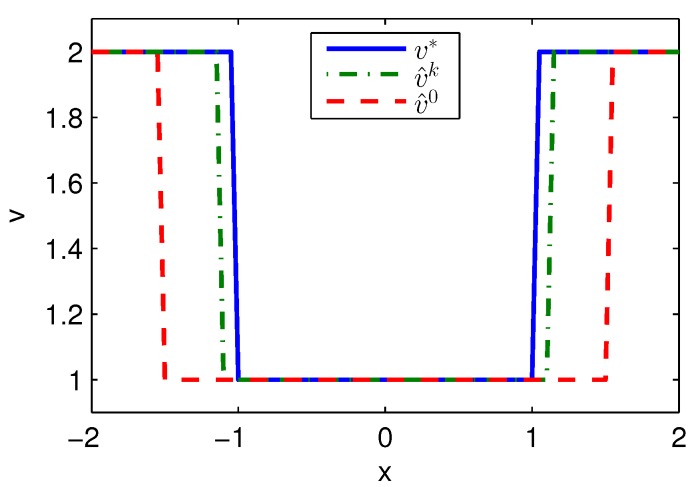
Switching policies achieved by Algorithm 2 applied in Example 1.

Apply Algorithm 2 and utilize the online state data from t=0 to 0.2 s. Then after calculation in 0.05 s, the approximate optimal cost is achieved with the corresponding approximate optimal switching policy through 6 iterations. The costs ${\hat{V}}^{0}$, ${\hat{V}}^{k}$ and $V^{*}$ are demonstrated in Figure 4. It can be obtained that compared with the initial cost of ${\hat{V}}^{0}$, the approximate optimal cost of ${\hat{V}}^{k}$ is close to the optimal cost of $V^{*}$. The state trajectories with $v^{0}$, ${\hat{v}}^{k}$ and $v^{*}$ applied in the system after t=0.25 s are demonstrated in Figure 5, where the state trajectory corresponding to ${\hat{v}}^{k}$ coincides with the one corresponding to $v^{*}$ and their largest error is 0.0155 while the largest error between the state trajectory corresponding to $v^{0}$ and the one corresponding to $v^{*}$ is 0.0563. The online trajectories also are too close to show the superiority of the proposed algorithm so that the switching policies $v^{0}$, ${\hat{v}}^{k}$ and $v^{*}$ when x∈[−2,2] are illustrated in Figure 6. It can be seen that ${\hat{v}}^{k}$ is approximate to $v^{*}$. The similarity rate of the schedules ${\hat{v}}^{k}$ and $v^{*}$ is 95.06% while the similarity rate of the schedules $v^{0}$ and $v^{*}$ is 75.31%.

**Example** **2.** *Consider a mass-spring-damper system as [21,22]:*

$$\begin{array}{c} {\dot{x}}_{1}\left(t\right)=x_{2}\left(t\right),\\ {\dot{x}}_{2}\left(t\right)=F_{v}-0.1x_{1}\left(t\right)-0.1x_{2}\left(t\right) \end{array}$$

*with v∈{1,2,3}, $F_{1}=1$, $F_{2}=-1$ and $F_{3}=0$. Here, the state $x_{1}\left(t\right)$ is the displacement of the mass measured from the relaxed length of the spring. $F_{v}$ is the external force acting on the mass. The initial state is x(0)=[2,2] and the function Q is*

$$Q\left(x\left(t\right)\right)=x{\left(t\right)}^{T}\left[\begin{array}{cc} 1 & 0\\ 0 & 1. \end{array}\right]x\left(t\right)$$

Choose the initial switching policy $v^{0}\left(x\right)=3$ when $\left|x_{1}\right|\leq 0.5$, $v^{0}\left(x\right)=2$ when $x_{1}>0.5$ and $v^{0}\left(x\right)=1$ when $x_{1}<-0.5$. The basis functions are polynomials with all possible combinations of the state variables up to the 4th degree without repetitions selected as [21,22]. Set the sample period δt=0.02 s.

**Figure 7:**
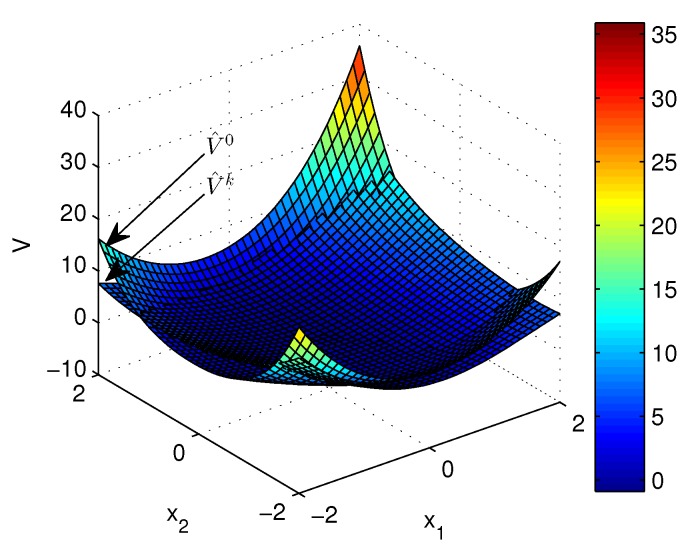
Costs achieved by Algorithm 1 applied in Example 2.

**Figure 8:**
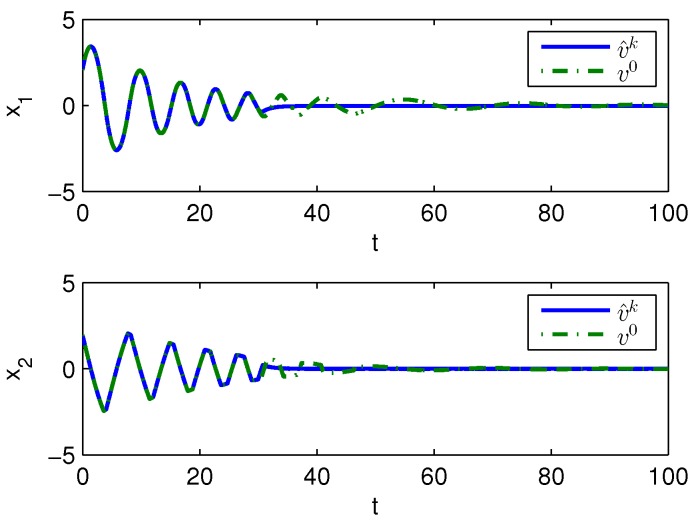
State trajectories with Algorithm 1 applied in Example 2.

**Figure 9:**
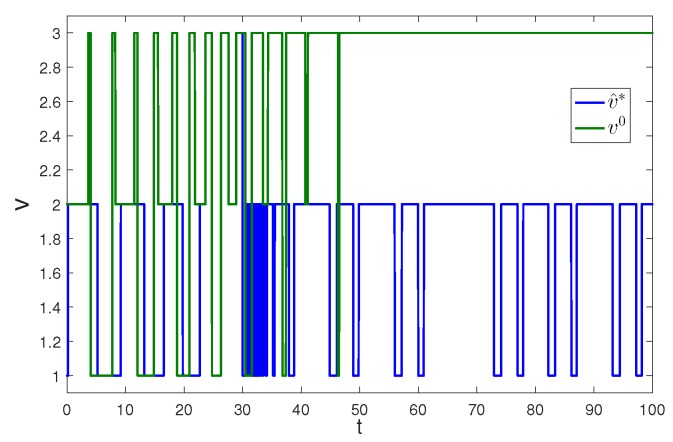
Switching policies with Algorithm 1 applied in Example 2.

Apply Algorithm 1 and utilize the online state data from t=0 to 23 s. Then after calculation in 7 s, the approximate optimal cost is achieved with the corresponding approximate optimal switching policy through 15 iterations. The initial cost ${\hat{V}}^{0}$ and the approximate optimal cost ${\hat{V}}^{k}$ are demonstrated in Figure 7. It can be seen that the approximate optimal cost ${\hat{V}}^{k}$ is less than the initial cost ${\hat{V}}^{0}$. The state trajectories with the initial switching policy $v^{0}$ and the approximate optimal switching policy ${\hat{v}}^{k}$ applied in the system after t=30 s are demonstrated in Figure 8, where the state trajectory corresponding to ${\hat{v}}^{k}$ converges to the origin quickly after t=30 s while the trajectory corresponding to $v^{0}$ converges slowly with decreasing oscillation amplitude. The corresponding switching policies $v^{0}$ and ${\hat{v}}^{k}$ are illustrated in Figure 9. It can be seen that $v^{0}$ which switches among three subsystems and finally stays at subsystem 3 results in that the trajectory corresponding to $v^{0}$ converges slowly with decreasing oscillation amplitude, while $v^{*}$ which switches between subsystem 1 and 2 results in that the state trajectory corresponding to ${\hat{v}}^{k}$ converges to the origin quickly after t=30 s.

**Figure 10:**
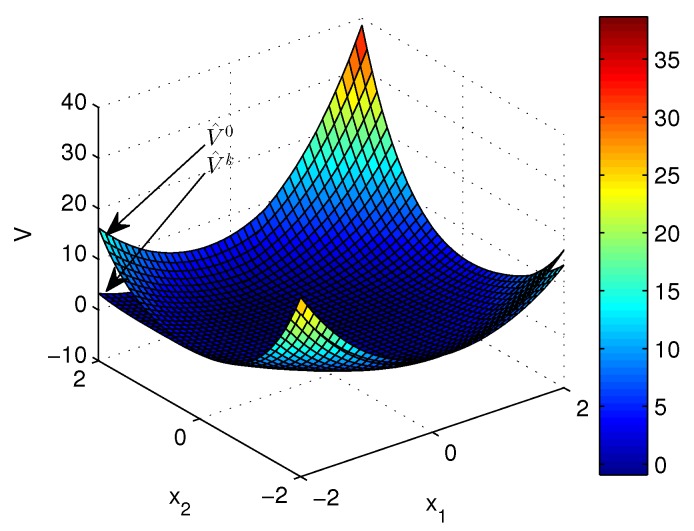
Costs achieved by Algorithm 2 applied in Example 2.

**Figure 11:**
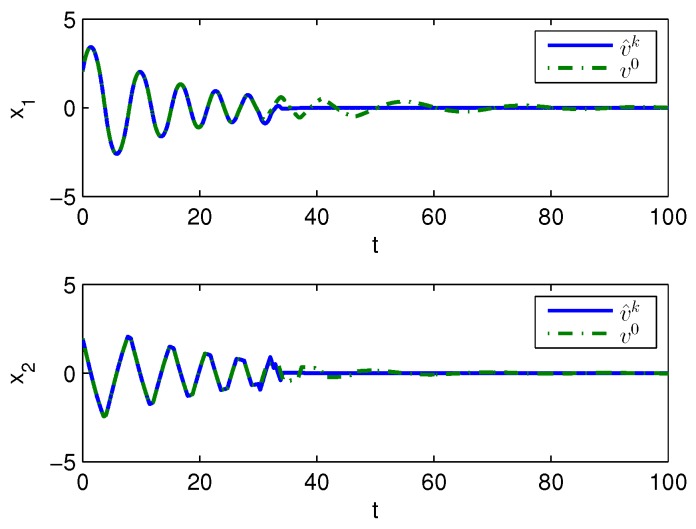
State trajectories with Algorithm 2 applied in Example 2.

**Figure 12:**
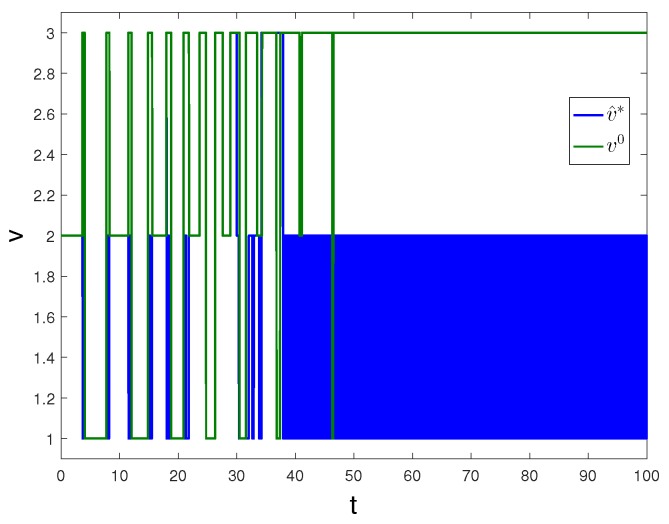
Switching policies with Algorithm 2 applied in Example 2.

Apply Algorithm 2 and utilize the online state data from t=0 to 23 s. Then after calculation in 7 s, the approximate optimal cost is achieved with the corresponding approximate optimal switching policy through 21 iterations. The costs ${\hat{V}}^{0}$ and ${\hat{V}}^{k}$ are demonstrated in Figure 10. It can be seen that the approximate optimal cost ${\hat{V}}^{k}$ is less than the initial cost ${\hat{V}}^{0}$. The state trajectories with $v^{0}$ and ${\hat{v}}^{k}$ applied in the system after t=30 s are demonstrated in Figure 11, where the state trajectory corresponding to ${\hat{v}}^{k}$ converges to the origin relatively quickly after t=30 s while the trajectory corresponding to $v^{0}$ converges slowly with decreasing oscillation amplitude. The corresponding switching policies $v^{0}$ and ${\hat{v}}^{k}$ are illustrated in Figure 12. It can be seen that $v^{0}$ which switches among three subsystems and finally stays at subsystem three results in that the trajectory converges slowly with decreasing oscillation amplitude, while $v^{*}$ which switches between subsystem 1 and 2 results in that the state trajectory converges to the origin relatively quickly after t=30 s.

**Remark** **4.** *In algorithm 2, the value of the initial switching policy $v^{0}\left(x\right)$ should include every element of V and there should exist enough data to calculate ${\hat{C}}_{i}^{0}$ for every i∈V so that the sampling stage must be long enough to ensure that.*

**Remark** **5.** *In the calculation, the fourth order Runge-Kutta algorithm is adopted to numerically evaluate the integrals which are necessary in certain terms such as $b(t_{r})$ and $d(t_{r})$.*

**Remark** **6.** *In Example 1, the proposed algorithms converge in 0.25 s while the online algorithm investigated in [38] requires more time. In Example 2, the proposed algorithms converge in 30 s while the online algorithms investigated in [21,22] require more time.*

In the two examples, the effectiveness of Algorithm 2 has been validated. The superiority of Algorithm 2 lies in that it can work for switched systems with unknown subsystems while Algorithm 1 can not work if the subsystems of switched systems are unknown. Practical examples of this kind of switched systems appear in a wide range of applications such as cyber-physical systems which embed software into the physical world and have proved resistant to modeling due to their intrinsic complexity arising from the combination of physical and cyber components and the interaction between them in [31]. Thereinto, data-driven research has been conducted for some specific examples such as complex electronics switching among low-voltage, middle-voltage and high-voltage models, and smart grid switching between base configuration and changed configuration. These examples require the data-driven approaches of Algorithm 2 where Algorithm 1 is inapplicable.

Simulation results show that Algorithms 1 and 2 can approximate the optimal solution effectively and efficiently. Algorithm 2 achieves the approximate optimal solution for unknown switched systems with infinite-horizon cost function, which has not been achieved well in existing literature as far as we know.

## 5. Conclusions

In this paper, an online PI-based algorithm inspired by the off-policy learning method and based on that, a data-driven PI-based algorithm, are proposed to approximate the optimal solution quickly for optimal scheduling problems. Only data produced by an initial switching policy is necessary and the approximation time is relatively short. The data-driven algorithm acquires useful knowledge of the weight vectors step by step through different data and solves the optimal scheduling problem for switched systems with unknown subsystems, only taking advantage of data. However, the dwell-time problems, which are important in practical applications, are not incorporated in this paper. So, optimal scheduling problems with dwell-time constraints will be investigated in the future.
